# Empirical comparison of deep learning models for fNIRS pain decoding

**DOI:** 10.3389/fninf.2024.1320189

**Published:** 2024-02-14

**Authors:** Raul Fernandez Rojas, Calvin Joseph, Ghazal Bargshady, Keng-Liang Ou

**Affiliations:** ^1^Human-Centred Technology Research Centre, Faculty of Science and Technology, University of Canberra, Canberra, ACT, Australia; ^2^Department of Dentistry, Taipei Medical University Hospital, Taipei, Taiwan; ^3^Department of Dentistry, Taipei Medical University-Shuang Ho Hospital, New Taipei City, Taiwan; ^4^3D Global Biotech Inc., New Taipei City, Taiwan

**Keywords:** fNIRS, biomarker, objective pain assessment, deep learning, machine learning

## Abstract

**Introduction:**

Pain assessment is extremely important in patients unable to communicate and it is often done by clinical judgement. However, assessing pain using observable indicators can be challenging for clinicians due to the subjective perceptions, individual differences in pain expression, and potential confounding factors. Therefore, the need for an objective pain assessment method that can assist medical practitioners. Functional near-infrared spectroscopy (fNIRS) has shown promising results to assess the neural function in response of nociception and pain. Previous studies have explored the use of machine learning with hand-crafted features in the assessment of pain.

**Methods:**

In this study, we aim to expand previous studies by exploring the use of deep learning models Convolutional Neural Network (CNN), Long Short-Term Memory (LSTM), and (CNN-LSTM) to automatically extract features from fNIRS data and by comparing these with classical machine learning models using hand-crafted features.

**Results:**

The results showed that the deep learning models exhibited favourable results in the identification of different types of pain in our experiment using only fNIRS input data. The combination of CNN and LSTM in a hybrid model (CNN-LSTM) exhibited the highest performance (accuracy = 91.2%) in our problem setting. Statistical analysis using one-way ANOVA with Tukey's (*post-hoc*) test performed on accuracies showed that the deep learning models significantly improved accuracy performance as compared to the baseline models.

**Discussion:**

Overall, deep learning models showed their potential to learn features automatically without relying on manually-extracted features and the CNN-LSTM model could be used as a possible method of assessment of pain in non-verbal patients. Future research is needed to evaluate the generalisation of this method of pain assessment on independent populations and in real-life scenarios.

## 1 Introduction

Pain assessment is of utmost importance, particularly in patients who are unable to communicate their pain level, often referred to as non-verbal patients. In clinical settings, self-reports using tools such as the visual analogue scale (VAS) or numerical rating scale (NRS) are widely regarded as the gold standard for determining pain levels in patients who are able to verbally report or express pain through writing or other means (Herr et al., 2011). However, it is important to note that in certain clinical conditions, patients may be unable to provide a self-report of their pain. These conditions include sedated patients with decreased levels of consciousness, critically ill patients receiving mechanical ventilation, individuals with impaired communication abilities, and older adults experiencing advanced dementia. In such cases, clinicians rely on a combination of behavioural and physiological indicators to infer and address pain in non-communicative patients. However, assessing pain using observable indicators can be challenging for clinicians due to the subjective nature of pain perception, individual variability in pain expression, and the potential for confounding factors that may influence the interpretation of these indicators. In order to address the challenges associated with using observable markers to assess pain, there is a need for a reliable tool that can assist clinicians in obtaining more objective pain assessments.

A method that has showed promising results to measure the physiological response associated with pain is functional near-infrared spectroscopy (fNIRS). fNIRS is a neuroimaging technique that measures brain activity by monitoring cerebral haemodynamics and oxygenation non-invasively. In particular, it measures cortical concentration changes in oxygenated haemoglobin (HbO) and deoxygenated haemoglobin (HbR) simultaneously, providing valuable information about brain activity (Rojas et al., 2016). fNIRS studies have showed that noxious stimuli in healthy and diseased subjects induce changes of oxygenation levels in different cortical regions (Ranger and Gélinas, 2014; Aasted et al., 2016; Fernandez Rojas et al., 2019). This neuroimaging tool has been previously employed by clinicians and scientists in different studies in neuroscience. In comparison with other neuroimaging methods like functional magnetic resonance imaging (fMRI), electroencephalography (EEG), and positron emission tomography (PET), fNIRS provides better temporal and spatial resolution compared to fMRI and EEG, respectively, and stands out for its safety profile, with no exposure to harmful ionising radiations, unlike PET. It is less susceptible to electrical noise since it uses optical sensors to record neural activity, compared to PET and EEG. In addition, fNIRS systems are small, lightweight, and highly portable, enabling research in diverse settings. This portability, unlike the bulky fMRI machines, allows fNIRS to be used in real-time applications (Fernandez Rojas et al., 2023). These attributes make this neuroimaging technique a potential candidate for real-world scenarios.

The use of machine learning algorithms has played a pivotal role in advancing fNIRS as a valuable tool in pain assessment. Machine learning refers to the set of methods of data analysis that can automatically detect patterns in data to make predictions or classify future data, to find structures such as clusters in the data, or to extract information to acquire new knowledge and make intelligent decisions (Lötsch and Ultsch, 2018). In pain research, machine learning and fNIRS have successfully been applied for detection and prediction of pain (Pourshoghi et al., 2016; Rojas et al., 2017c, 2021; Gökçay et al., 2018). In these examples, machine learning leverages pain-related data to construct a feature mapping, enabling the identification or prediction of new data by establishing a distinctive pain signature. However, one limitation of machine learning models is their dependence on hand-crafted feature extraction, which necessitates human intervention and prior domain knowledge to carefully design and select the most relevant features for effective task-solving by the learning models. Although this approach has proven to be very effective in many applications, this can also limit the model performance. Therefore, it would be desirable to make learning algorithms less dependent on hand-crafted features, so that these systems could be less subjective, less labour intensive, and more efficient (Bengio et al., 2013).

Deep learning has gained popularity in recent years motivated by its success in various classification tasks and applications. For instance, in computer vision (Krizhevsky et al., 2012), image classification (Szegedy et al., 2015), natural language processing (Sutskever et al., 2014), and automatic speech recognition (Hinton et al., 2012). An advantage of deep learning methods is the automatic feature learning from data, which largely contributes to improvements in model performance. Learned features are extracted automatically to solve a specific task, which avoids the level of subjectivity and domain knowledge in the design of hand-crafted features by learning from raw input data (Plis et al., 2014). Such algorithms develop a multi-layered, hierarchical structure of learning and representing data (features), where higher-level features are defined in terms of lower-level features (Najafabadi et al., 2015). The hierarchical learning structure in deep learning algorithms aims to emulate the deep, layered learning process of the neocortex, which processes sensory input in the human brain. This involves automatically extracting increasingly complex features until objects are recognised (Hawkins et al., 2019). This high-level of abstraction offers more efficient and sophisticated features that often outperform models that use hand-crafted features.

Several deep learning architectures have been proposed for time series modelling that can be applied to fNIRS data. In particular, Convolutional Neural Networks (CNNs) have produced the greatest impact in many domains since AlexNet (Krizhevsky et al., 2012) won the ImageNet competition in 2012. Thanks to this success, CNNs have been adopted for time series analysis in many different applications. Initially designed for 2D (width and height) image analysis, CNNs can be used to analyse time series data by analysing the time varying signals with 1D convolutions (Rim et al., 2020). A convolution operation refers to the inner product (multiplication by element and then summation) for different windowed data and a kernel matrix (also called a filter) (Jin et al., 2020). In this case, the convolution process is seen as a sliding window filter applied over the time series to extract temporal relationships (Lim and Zohren, 2021). The filter can also be seen as a generic non-linear transformation of a time series (Fawaz et al., 2019). The same set of filter weights at each time step can be used (weight sharing) since temporal CNNs are built under the assumption that relationships are time-invariant across all the time series. This property allows CNNs to learn filters that are invariant across the time dimension.

Another popular type of architecture is Recurrent Neural Network (RNN), which has been used extensively in temporal forecasting applications (Rangapuram et al., 2018). An RNN architecture contains an internal memory state that provides the output of the new data based on the present and the recent past; for this reason, these architectures are referred as having memory. This is useful in applications where the output depends on previous computations, e.g., text analysis, speech, and DNA sequences (Rav̀ı et al., 2016). However, this model is not suitable for tasks that require learning long-range dependencies in the data, as the difficulty with long-term dependencies arises from the exponentially small or large weights given to long-term interactions—an issue described as the vanishing and exploding gradient problem (Pascanu et al., 2013). An architecture that addresses this problem is the Long Short-Term Memory (LSTM) networks. These networks use a memory cell to store long-term information, regulated through a series of gates (input, output, and forget gates) that control the flow of information, and allow gradient flow within the network (Rav̀ı et al., 2016). This architecture is based on the idea of creating paths through time that have derivatives that do not vanish by accumulating information (such as evidence for a particular feature or category) over a long duration, and once this information is employed, the neural network can forget the old state (Goodfellow et al., 2016). LSTMs have the ability to remember patterns for long time series, a useful property in physiological signal analysis.

Another architecture that explores the complementarity of CNNs and LSTMs for time series modelling is a hybrid CNN-LSTM model. This architecture considers the benefits of both models, as CNNs are good at extracting spatial relationships while reducing frequency variations, and LSTMs are good at identifying short-term and long-term dependencies (Ordóñez and Roggen, 2016). In this architecture, the conventional LSTM network is extended by adding a feature extraction phase using convolutions, which potentially enhances the prediction capability of the LSTM network (Yan et al., 2018). In addition, the use of better features captured by the CNN layers has showed to improve the performance of LSTMs in speech recognition tasks, when these model have been used as two separated models (Sainath et al., 2015). In addition, the CNN-LSTM architecture has showed more accurate and robust results in comparison with other architectures in different time-series classification and regression tasks such as, EEG-based user identification (Sun et al., 2019), wrist kinematics estimation using electromyography (EMG) (Bao et al., 2020), fall detection using accelerometer and gyroscope data (Nait Aicha et al., 2018), and emotion recognition using heart rate and galvanic skin response (Kanjo et al., 2019).

In this paper, an empirical comparative study of deep learning approaches for the objective assessment of pain is presented. The motivation to utilise deep learning models was to investigate their potential superiority for classifying different levels of pain without the need of feature engineering. This paper presents the following novelties with respect to the current literature: (1) we demonstrated that the use of deep learning for the identification of different pain intensities using fNIRS data without the need of feature engineering; (2) we provided a comparison between classical machine learning and state-of-the-art deep learning models, based on time series classification tasks; and (3) We also showed that the hybrid model composed of a convolutional neural network (CNN) and long short-term memory (LSTM) model (namely CNN-LSTM) achieved the best performance in our problem setting, while the other independent deep learning models (CNN, LSTM) achieved remarkable results as well. Finally, this study aims to contribute to the development of an objective pain assessment method that will benefit patients who cannot provide a self-report of pain verbally, in writing, or by other means. Future research will evaluate the generalisation of this method of pain assessment on independent populations and in real-life scenarios.

## 2 Methodology

### 2.1 Participants

Eighteen healthy adults (15 males, 3 females) with a mean age of 31.9 years participated in the experiments. To maintain consistency in functional responses attributed to brain lateralisation, all participants were right-handed. Prior to the initiation of the experiments, informed consent was obtained from each participant. Individuals with a history of significant medical disorders, current unstable medical conditions, or those currently taking medication were excluded from participation. The experiments were designed through collaboration between the School of Oral Medicine at Taipei Medical University (TMU, Taiwan) and the University of Canberra (UC, Australia), adhering to the principles outlined in the Declaration of Helsinki guidelines. Approval for this study was granted through a thorough review process by the TMU Joint Institutional Review Board under contract number 201307010.

### 2.2 fNIRS system

**Figure 1:**
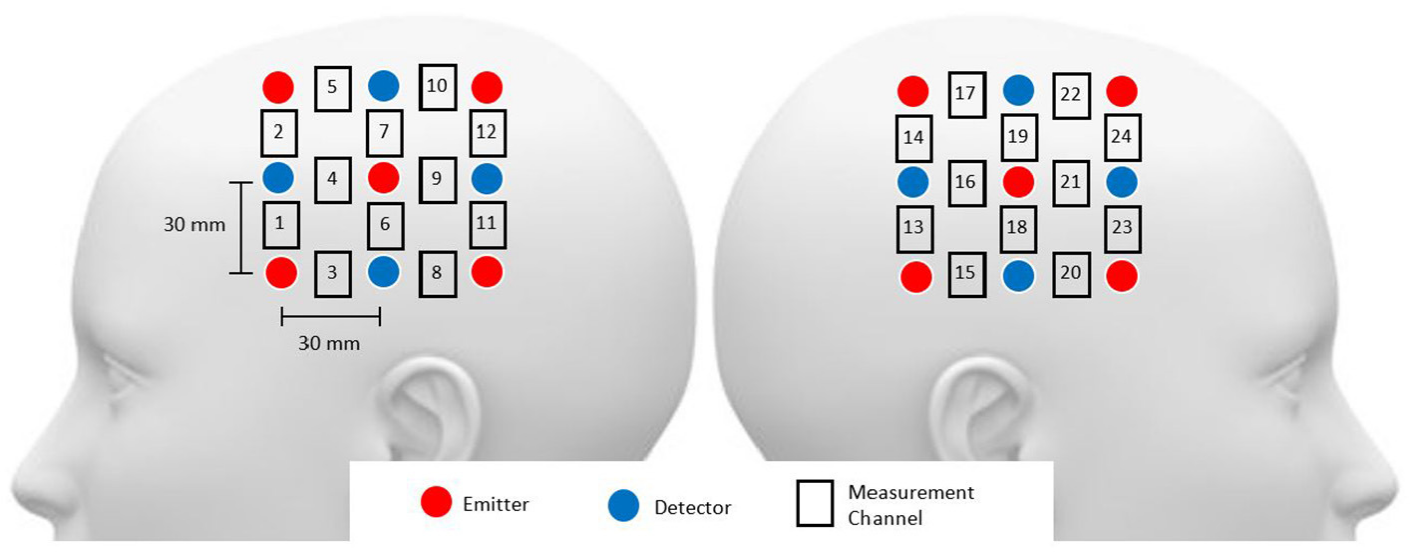
Head probe arrangement included channels 1–12 over the right hemisphere and channels 13–24 over the left hemisphere, with probes around the C3 and C4 areas. The source-detector distance was maintained at 3 cm.

Functional NIRS recordings were obtained using the Hitachi ETG-4000 multichannel optical topography system (Hitachi Medical Corporation, Tokyo, Japan). This system employs two near-infrared light wavelengths (695 nm for oxygenated haemoglobin [oxy-Hb] and 830 nm for deoxygenated haemoglobin [deoxy-Hb]) to measure haemoglobin concentration changes in the cerebral cortex. The spectrometer featured a 24-channel cap, organised into 12 channels per hemisphere (refer to Figure 1). Each hemispheric probe comprised five sources (red circles) and four detectors (blue circles), providing a total of twelve source-detector pairs. Following the EEG 10–20 system, the measurement probes were centred on the C3 and C4 positions (Rojas et al., 2017b). For the purposes of this study, only the oxy-Hb signals were utilised due to their superior signal-to-noise ratio compared to deoxy-Hb signals (Rojas et al., 2017a). The sampling rate for data acquisition was set at 10 Hz.

### 2.3 Pain stimuli

The stimulation protocol followed the quantitative sensory testing (QST) protocol using the threshold and tolerance of pain (Rolke et al., 2006). The protocol defines a pain threshold (low pain) as the lowest stimulus intensity at which stimulation becomes painful, and pain tolerance (high pain) as the highest intensity of pain a person can endure. Using a sensory analyser (Pathway CHEPS, Medoc Ltd., Israel), participants were exposed to gradually increasing or decreasing temperatures with a thermode (Rolke et al., 2006), while fNIRS was recorded simultaneously. The thermode administers both heat and cold to the skin, with a contact area of 9.0 *cm*^2^ and a baseline temperature set at 32^*o*^C. Pain measurements were conducted on the back of the left hand, where participants pressed a button upon experiencing pain (threshold test) and the highest intensity of pain (tolerance test). The temperature of the thermode at the point of becoming painful or unbearable was recorded as the thermal pain threshold or thermal pain tolerance, respectively. The use of a thermode increased the consistency of each test as it represents a set of standard stimuli applied to all the participants, as compared to other methods (e.g., cold pressor test) that might introduce considerable variability in the experimental conditions (McIntyre et al., 2020).

**Figure 2:**
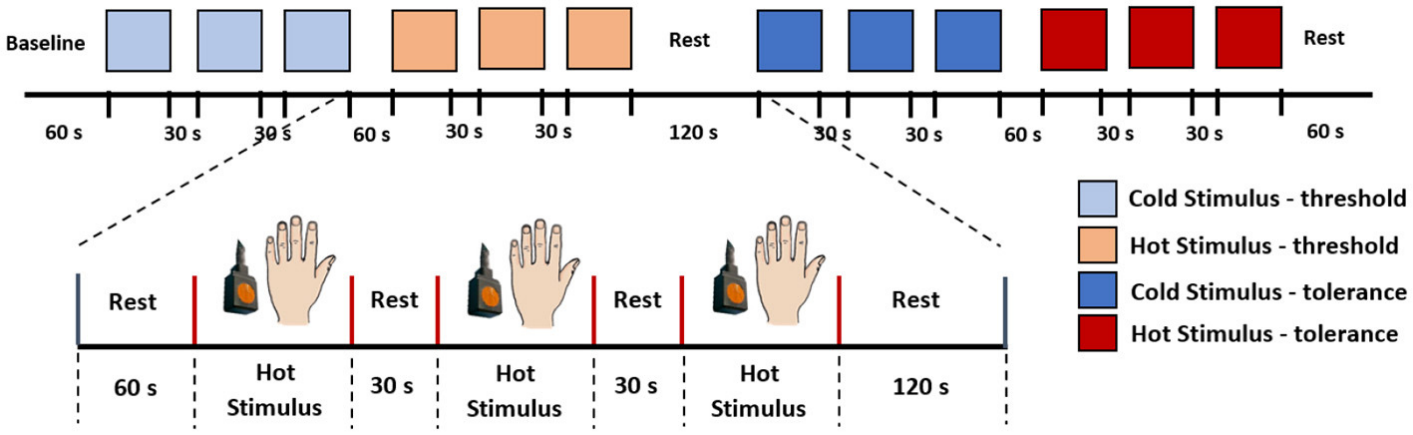
Stimulation paradigm. In this example, we initiated the experiment with the pain threshold test, followed by the pain tolerance test. Each subject underwent both cold and hot stimuli applications on the back of their hand, and the sequence of stimuli was randomised for each participant.

The experiment comprised two tests: the thermal pain threshold (low pain) and the thermal pain tolerance (high pain), with a 2-minute rest between each test. Baseline data were measured during the initial 60 seconds of the experiments at rest. Following this, the stimulation was randomly applied between the threshold and tolerance tests. Figure 2 illustrates the stimulation paradigm. In this example, three consecutive measurements of pain threshold for cold and heat are obtained, with a 60-s rest between cold and heat detection and a 30-s rest between each repetition of the same stimulus. Based on these measurements the fNIRS data were organised into four categories for classification: (0) Low-Cold (low pain), (1) Low-Heat (low pain), (2) High-Cold (high pain), and (3) High-Heat (high pain). These categories (0–3) were used to label the database and used as classes for the classification task. Therefore, each participant completed 4 thermal tests (repeated 3 times for low-cold, low-heat, high-cold, and high-heat, respectively); in total, 216 trials were obtained as our dataset, with 54 trials for each type of thermal test.

In our experimental paradigm, we exposed participants to multiple types of stimuli (i.e., cold and heat). The rationale to incorporate various stimuli was to emulate the diversity of pain sources encountered in real-world scenarios. Pain experiences in everyday life surpasses simple variations in intensity, encompassing dimensions that span thermal, electrical, or mechanical aspects, among others. Our experimental design sought to capture some of this variability by exposing participants to a range of stimuli, creating a more authentic representation of pain processing under varied conditions. The incorporation of diverse stimuli becomes relevant when considering the activation of different pain receptors. For instance, cold stimuli predominately engage cold receptors (TRPM8), while hot stimuli activate heat receptors (TRPV1) (Takaishi et al., 2016). This inclusion of diverse stimuli was aimed at developing a robust model capable of generalising across various types of pain, within our experimental conditions. This approach enhances the practical utility of our model, especially in real-world pain assessment scenarios where pain can manifest through a multitude of sensory channels.

### 2.4 fNIRS pre-processing

**Figure 3:**
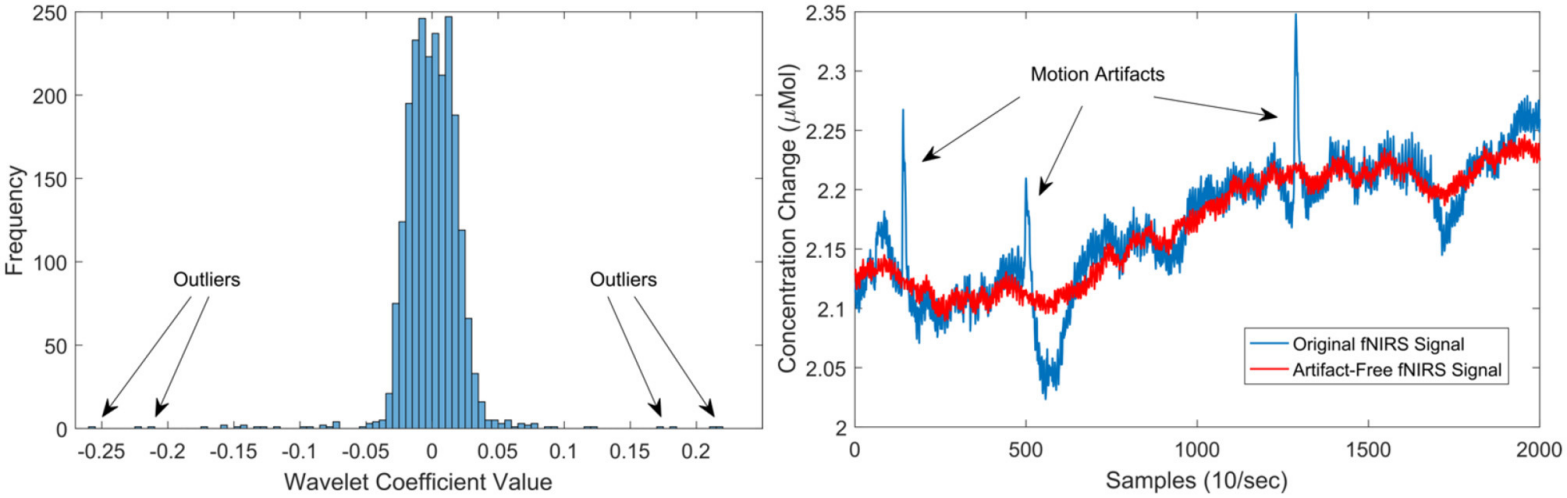
**Left** illustrates the distribution of wavelet coefficients for an fNIRS signal, showcasing the impact of motion artefacts on the distribution. On the **right**, the outcomes of the motion artefact algorithm are presented. The blue signal represents the original signal with three prominent moving artefacts (spikes), while the red signal depicts the artefact-free signal post the application of the de-noising procedure.

Although, deep neural networks can accept as input raw sensor signals, motion artefacts in fNIRS data severely affect the quality of the signals. Therefore, motion artefacts were removed using the discrete wavelet transform (DWT) (Molavi and Dumont, 2012; Rojas et al., 2017a). Motion artefacts are observed as spikes in the amplitude of the fNIRS data with several orders of magnitude larger than the expected variance of the signals. This behaviour can be used to detect and eliminate motion artefacts in the fNIRS data. Following this property, motion artefacts can be determined by identifying those wavelet coefficients that do not belong to the probability distribution (i.e., outliers) of the fNIRS captured data. However, not all large wavelet coefficients are motion artefacts, thus, a two-tailed Student's *t*-test was used to evaluate (with *p* < 0.05) the resulting wavelet coefficients. The identified coefficients and their corresponding frequencies are then eliminated in the wavelet domain, leaving the remaining frequencies intact. Thus, a fNIRS signal can be free of motion artefacts using this method. A clear advantage of using this method is that the signal length remains intact and no data is lost in this process. For more information regarding the motion artefact removal method, the interested reader is referred to Molavi and Dumont (2012). In total, four subjects presented motion artefacts. Figure 3 presents an example of the cleaning procedure to remove moving artefacts from the fNIRS signals.

The final step in the pre-processing stage involved a normalisation procedure and the partition of the fNIRS data into consecutive segments for the analysis. First, the data was normalised using the z-score normalisation, and scaled to a range of 0–1 per subject. This procedure was done to ensure an equal contribution of all variables to the classification process and thus, prevent that variables with greater values influence the classification results (Horst et al., 2019). In addition, the scaling process prevents any numerical problem that might affect the learning and convergence of the networks (Bishop et al., 1995). Secondly, a sliding window approach was used to split the multiple channels into small segments that can be processed by both the classical machine learning and deep learning models (Creagh et al., 2022). The window size was obtained empirically, windows of different lengths (10, 20, 30, 40, 50, and 60 s) were tested; with the 30-s window size producing the highest accuracy among all classifiers using all data. Therefore, 300 samples (10Hz sample rate) per window were used for the remaining of the analysis for both the reference models and deep learning models. After the pre-processing stage, the fNIRS signals are used to extract features for traditional machine learning models, while they are directly input into the deep learning models for analysis.

### 2.5 Learning models

This study proposes the use of deep learning to eliminate the need for manual feature engineering in the classification of pain with fNIRS data. In particular, we tested three of the most successful deep learning models for time series classification (Fawaz et al., 2019; Wang et al., 2020; Lim and Zohren, 2021), these are: convolutional neural network (CNN), long short-term memory (LSTM), and CNN-LSTM. As CNNs are good at identifying spatial relationships and LSTMs are good at temporal modelling, it is hypothesised that an unified architecture (CNN-LSTM) will take advantage of each network's properties to produce better results (Sainath et al., 2015). In order to test and compare the proposed models, two experiments were carried out. The first experiment was designed to obtain reference values using classical machine learning models by obtaining popular hand-crafted features as baseline models. In the second experiment, the CNN, LSTM, and CNN-LSTM models are evaluated separately without any crafting in feature engineering.

#### 2.5.1 Reference models

In order to characterise the benefits of the proposed deep learning models, baseline models were implemented. These models are based on well-know linear and non-linear machine learning classifiers. The linear classifiers are linear discriminant analysis (LDA), logistic regression (LR), and linear support vector machines (LSVM); while the non-linearc neighbours (KNN), random forest (RF), and Gaussian support vector machines (GSVM). The hyperparameters were optimised using Bayesian optimisation for each classifier, which will be described in detail in the next subsections. These parameters are the regularisation method [*l*1, *l*2, *none*] and the regularisation strength *C* [0.001, 0.01, 0.1, 1, 10, 100] for LR, the regularisation parameter C [0.01, 0.1, 1, 10, 100] for both the LSVM and GSVM, additionally the kernel parameter γ [0.0001, 0.001, 0.01, 0.1] for GSVM, the number of trees [50–500], maximum tree depth [10–100], and the number of features considered at each split [*auto*, *sqrt*] for RF, the number of neighbours [3–30] for KNN, and for the LDA classifier no parameters were optimised as LDA presents a closed-form solution (Sifaou et al., 2020). The parameters that showed the highest validation accuracy (refer to Figure 5) were selected as the optimal parameters.

The hand-crafted features are based on popular statistical measures: mean, standard deviation, auto correlation, kurtosis, skewness, floor (min), and ceiling (max) values. In total 168 features (24 channels × 7 features) were obtained. Finally, principal component analysis (PCA) was implemented to reduce the number of features, computational complexity, and possible overfitting; based on the scree plot, the top 8 PCs were selected and used for classification.

#### 2.5.2 Proposed models

For comparison purposes, the individual models (CNN and LSTM) shared a similar architecture with the combined model (CNN-LSTM). This is done with the idea that differences in performance between the individual and combined models result from architectural differences rather than better pre-processing or *ad hoc* customisations (Ordóñez and Roggen, 2016). Similar to the reference models, parameter optimisation was carried out using Bayesian optimisation. The parameters common to the proposed models are learning rate [0.0001, 0.001, 0.01], batch size [32, 64, 128], L2 weight regularisation [0.1, 0.2, 0.3], number of epochs [100, 200, 300, 400, 500, 1,000], and optimiser [Adam, RMSprop, SGD].

The architecture of the CNN implemented in this study has the following components. Two 2D convolutional layers (i.e., Conv2D) were considered in this architecture, the convolutional layers included filters with kernel size {3 × 3, 5 × 5} with ReLU activation function. These kernel sizes allow the network to capture both the temporal changes and the inter-channel interactions. The first convolution layer has 64 filters and the second convolution layer has 32 filters, the convolution kernels have a stride of 1 and padding of 1. After the convolutional layers, a max-pooling layer with a pool size of 2, stride of 1, and padding of 1 was implemented. Then, a dropout layer with a dropout rate of 30% to reduce over-fitting to the training data (Srivastava et al., 2014). After that, a flatten layer and a dense fully connected layer with 30 neurons were implemented. Finally, the output layer gives the final probabilities for each label using Softmax as activation function.

The LSTM model was implemented using a single LSTM recurrent layer with 100 hidden units. The reason to use a single LSTM layer is because LSTMs are prone to over-fitting more easily than conventional recurrent networks. Deeper networks can capture more complex patterns but might also be prone to overfitting, especially with limited data (Bao et al., 2020). A hyperbolic tangent (tanh) activation function was used in the LSTM layer. After the LSTM layer, a dropout layer with 30% dropout rate was adopted. Then, the output of the LSTM layer is flattened and fed into a fully connected layer with 50 neurons and ReLU activation. The final layer is another fully connected layer with four nodes and Softmax activation for classification.

**Figure 4:**
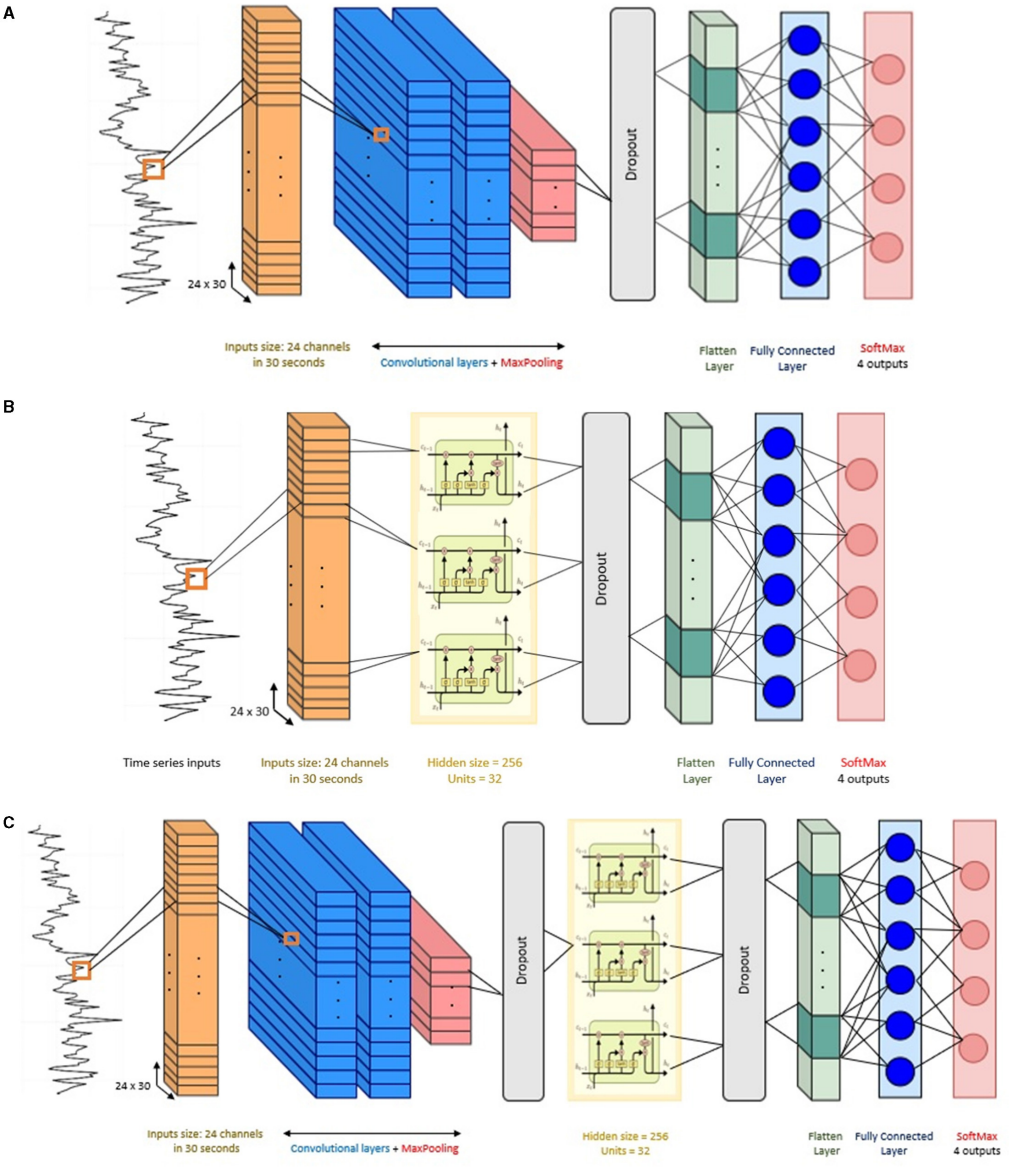
Network structure of the baseline models. **(A)** Convolutional neural network (CNN), **(B)** long short-term memory (LSTM), and **(C)** CNN-LSTM architecture.

In the hybrid mode, the CNN-LSTM has a similar architecture as the individual CNN and LSTM models described above. The output data from the CNN layers serve as input to the LSTM layer. Thus, the number of layers remained the same, with two convolutional layers and a single LSTM layer. To maintain the temporal nature of the data expected by the LSTM layer, a TimeDistributed wrapper was applied to each layer of the CNN model. Overall, the architecture of this CNN-LSTM is composed of: two convolution layers followed by a maxpooling layer and a dropout layer, a flatten layer to format the data into the shape required by the LSTM, then a LSTM layer followed by dropout layer and flatten layer, a fully connected layer, and finally another fully connected layer as output layer to make predictions using Softmax activation. Figure 4 presents the architecture of the compared models in this study.

### 2.6 Optimisation of learning models

To discover the optimal combination of various options that need to be explored, a grid search can be performed to explore every possible combination. However, this approach can become computationally intensive due to a combinatorial explosion (Haddad et al., 2020). To address this challenge, we utilised a Bayesian optimisation approach using the Optuna framework, to reduce computational complexity (Akiba et al., 2019). Optuna utilises the Tree-structured Parzen Estimator (TPE) by default, known for its efficiency and significantly lower computational cost compared to a grid search method (Bergstra et al., 2011). The number of trials was kept to the default (100 trials) for all models. An automatic early stopping (i.e., pruning) for unpromising trials was employed to reduce optimisation time. A pruner using the asynchronous successive halving algorithm was used with default parameters. Given the balanced distribution of the dataset across all classes, the optimisation objective focused on maximising accuracy throughout all trials and models.

**Figure 5:**
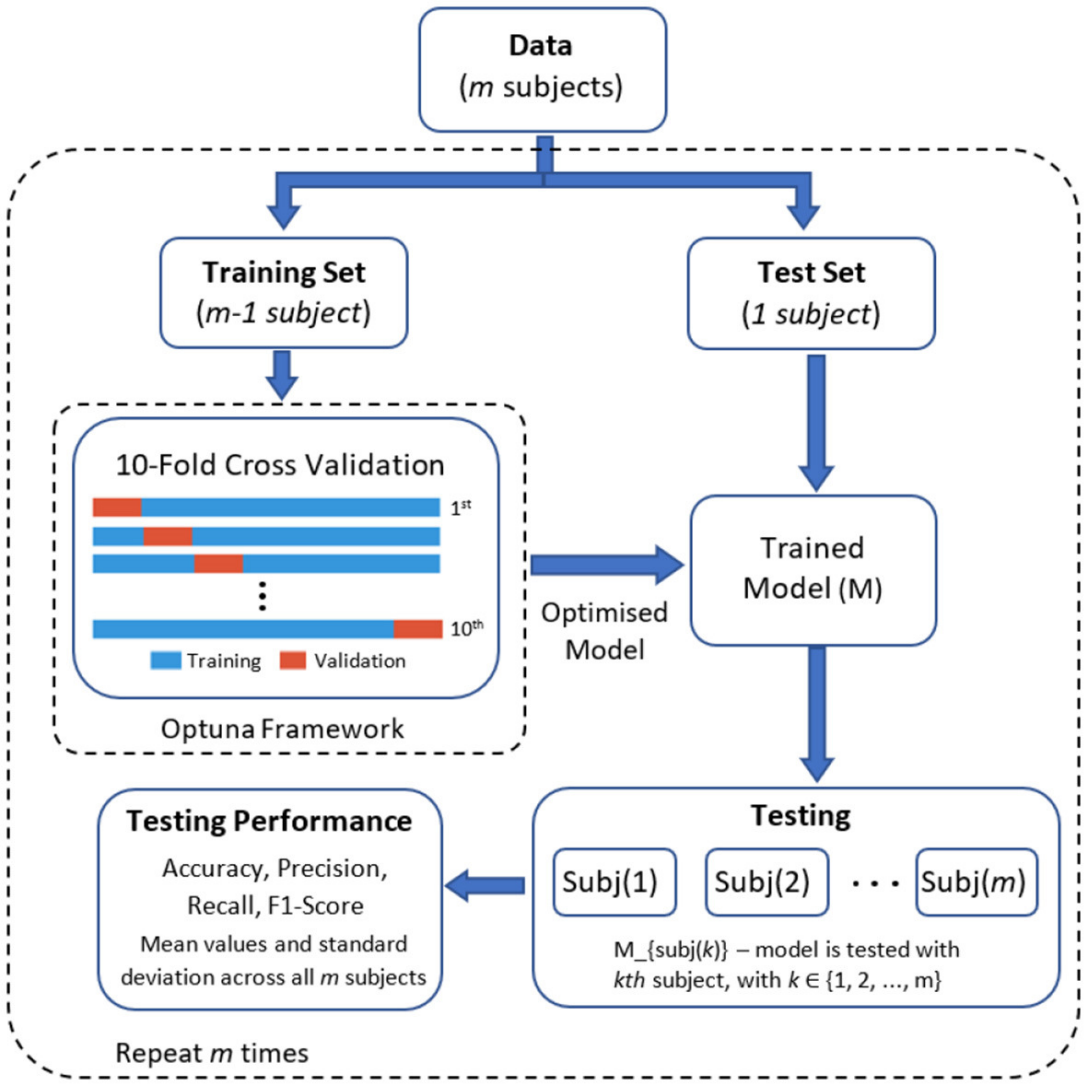
Graphical representation of the optimisation and classification process.

The parameter optimisation process was completed using an inner 10-fold cross validation within the training set. In this process, the dataset is divided into 10 equal parts (folds), nine folds are used for training the model with different sets of hyperparameters and the remaining one fold to evaluate the model's performance. This process is repeated for all 10 folds, ensuring that each fold serves as the test set once. After completing the 10 iterations of training and evaluation, the model with the best average accuracy is selected as the final optimised model. This final model with the best parameter was applied to the test set (i.e., the subject left out in the current iteration). Figure 5 presents the graphical representation of this process.

### 2.7 Model evaluation

**Figure 6:**
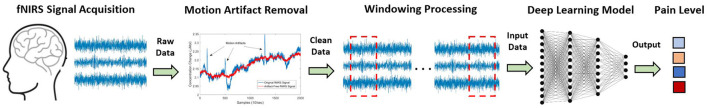
Overview of data acquisition and data analysis process. The subject is stimulated with heat or cold stimuli while fNIRS data is recorded simultaneously. The fNIRS data is cleaned of motion artefacts. Then, the data is segmented in overlapping windows of 30 s each. The deep learning models are fed with the windowed data to make a prediction based on that. Finally, a level of pain is obtain (low, high) from the two different stimuli (cold, heat) is obtained as output from the deep learning models.

We employed a leave-one-subject-out cross-validation approach using the optimised parameters for each model. In this method, the learning models were trained with the data of 17 participants and then tested with the data of the remaining participant. This process was iteratively repeated until all subjects had been included in the testing dataset (refer to Figure 5 for a visual representation). Four standard performance metrics were used to compare the deep learning models. These metrics are: accuracy, precision, recall, and F1 score. These metrics are based on the confusion matrix and under one-vs.-all multiclass classification approach with four classes (*k* = 4): (1) low-cold pain, (2) low-heat pain, (3) high-cold pain, and (4) high-heat pain. Table 1 provides a summary of each metric, where *P* and *N* denote the number of positive and negative samples, respectively. True Positive (TP) represents the count of correctly predicted samples belonging to the positive class, True Negative (TN) signifies the number of correctly predicted samples in the negative class. False Positive (FP) indicates the count of incorrectly predicted samples in the positive class (Type I error), while False Negative (FN) refers to the number of incorrectly predicted samples in the negative class (Type II error) (Tharwat, 2020). In our multi-class problem the macro-average for all metrics was computed, the macro-average computes the metric treating all classes equally (each class has the same weight in the average) since all classes have the same number of samples, and then take the arithmetic mean of each individual class (Sokolova and Lapalme, 2009). Finally, Figure 6 presents an overview of the overall methodology used in this study.

**Table 1:** Performance metrics using macro-averaging for the multi-class classification problem in this study.

**Metric**	**Formula**	**Note**
		
*Accuracy*	$\frac{\overset{k}{\underset{i=1}{\sum }}\frac{TP_{i}+TN_{i}}{TP_{i}+FN_{i}+FP_{i}+TN_{i}}}{k}$	The average per-class effectiveness of a classifier.
		
*Precision_*M*_*	$\frac{\overset{k}{\underset{i=1}{\sum }}\frac{TP_{i}}{TP_{i}+FP_{i}}}{k}$	The average per-class agreement of the data labels with the positive labels given by the classifier.
		
*Recall_*M*_*	$\frac{\overset{k}{\underset{i=1}{\sum }}\frac{TP_{i}}{TP_{i}+FN_{i}}}{k}$	Effectiveness of a classifier to identify positive labels.
*F1-score_*M*_*	$\frac{2*Precision_{M}*Recall_{M}}{Precision_{M}+Recall_{M}}$	Relations between data's positive labels and those given by a classifier.

Where *k*, total number of classes; *i*, individual class; *TP*, true positive; *TN*, true negative; *FP*, false positive; *FN*, false negative; *M*, macro-averaging.

In addition, a statistical hypothesis test was implemented on accuracies of the learning models. One-way analysis of variance (ANOVA) was used to compare the classifiers and Tukey's test as *post-hoc* test. A *p* value that is >0.05 was not considered statistically significant. The statistical test is designed to evaluate if the models perform equally well and to determine if the difference is statistically significant. Therefore, the null hypothesis of this test is: *H*_*o*_: *The mean accuracy value is the same across all classifiers*. In other words, if the null hypothesis is retained (reject = FALSE) in the comparison between the proposed models and baseline models, it will indicate not only that baseline models are at most as accurate as the proposed models, but also that there are not significant differences between the performance of the models.

## 3 Results

This paper explores the use of deep learning for the objective assessment of pain using fNIRS data. In order to characterise the benefits of the proposed deep learning models, three baseline models (LDA, LR, SVM) were implemented, using well-established statistical hand-crafted features. First, this section provides the temperature readings during the experiment. Then, the accuracy scores and other performance metrics from both the baseline models and the proposed methods are presented.

### 3.1 Threshold and tolerance of pain

**Figure 7:**
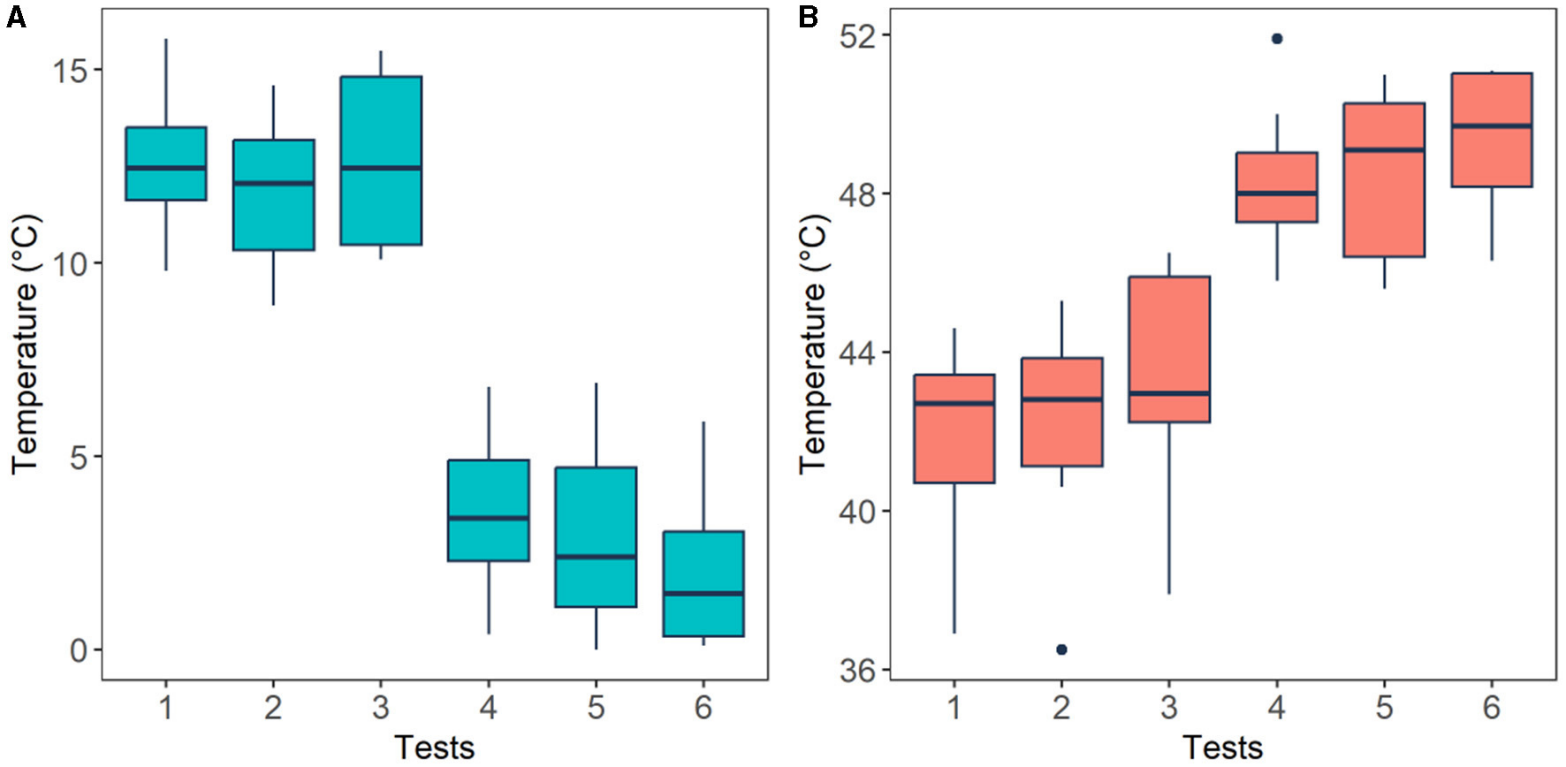
Thermal threshold (low pain) and tolerance (high pain) levels perceived by the participants after cold **(A)** and heat **(B)** stimuli. Horizontal black lines are the median values across all participants for each test. Pain threshold (tests 1–3) and pain tolerance (tests 4–6).

Subject's pain perception was obtained using the thermal threshold and tolerance test following the QST protocol. Using this method four temperature readings were obtained from each participant (please refer to Section 2.3), these are: pain threshold (low pain) and pain tolerance (high pain) of cold and heat stimuli. The averaged temperature readings from each stimulus are presented in Figure 7. The plot presents temperature recordings of threshold (Tests 1-3) and tolerance (Test 4–6) of cold (Figure 7A) and heat (Figure 7B) stimuli across all participants. Median temperature recordings (± standard deviations) from pain thresholds (initial pain sensation) of cold (12.45 ± 1.97,12.05 ± 1.93, 12.45 ± 2.22°C) and hot stimuli (42.70 ± 2.44, 42.80 ± 2.75, 42.95 ± 2.92°C) exhibited smaller temperature values than those from pain tolerance (highest pain sensation) of cold (3.40 ± 2.07, 2.40 ± 2.71, 1.45 ± 2.12°C) and heat stimuli (48.00 ± 1.92, 49.10 ± 2.11, 49.70 ± 1.82°C). These four levels were used to define the four classes for the classification task, which correspond to the two types of pain (cold and heat) at two levels of pain (low and high).

In order to validate experimental conditions, the temperature readings were analysed employing a Wilcoxon signed-rank test. Figure 7 illustrates that, as expected, cold pain tolerance corresponds to lower temperatures, while hot pain tolerance corresponds to higher temperatures. A significant difference (*p* < 0.05) was found between cold pain threshold and tolerance (*p* = 0.0018), as well as hot pain threshold and tolerance (*p* = 0.002), affirming that experimental conditions align with anticipated responses.

### 3.2 Performance of the reference models

**Figure 8:**
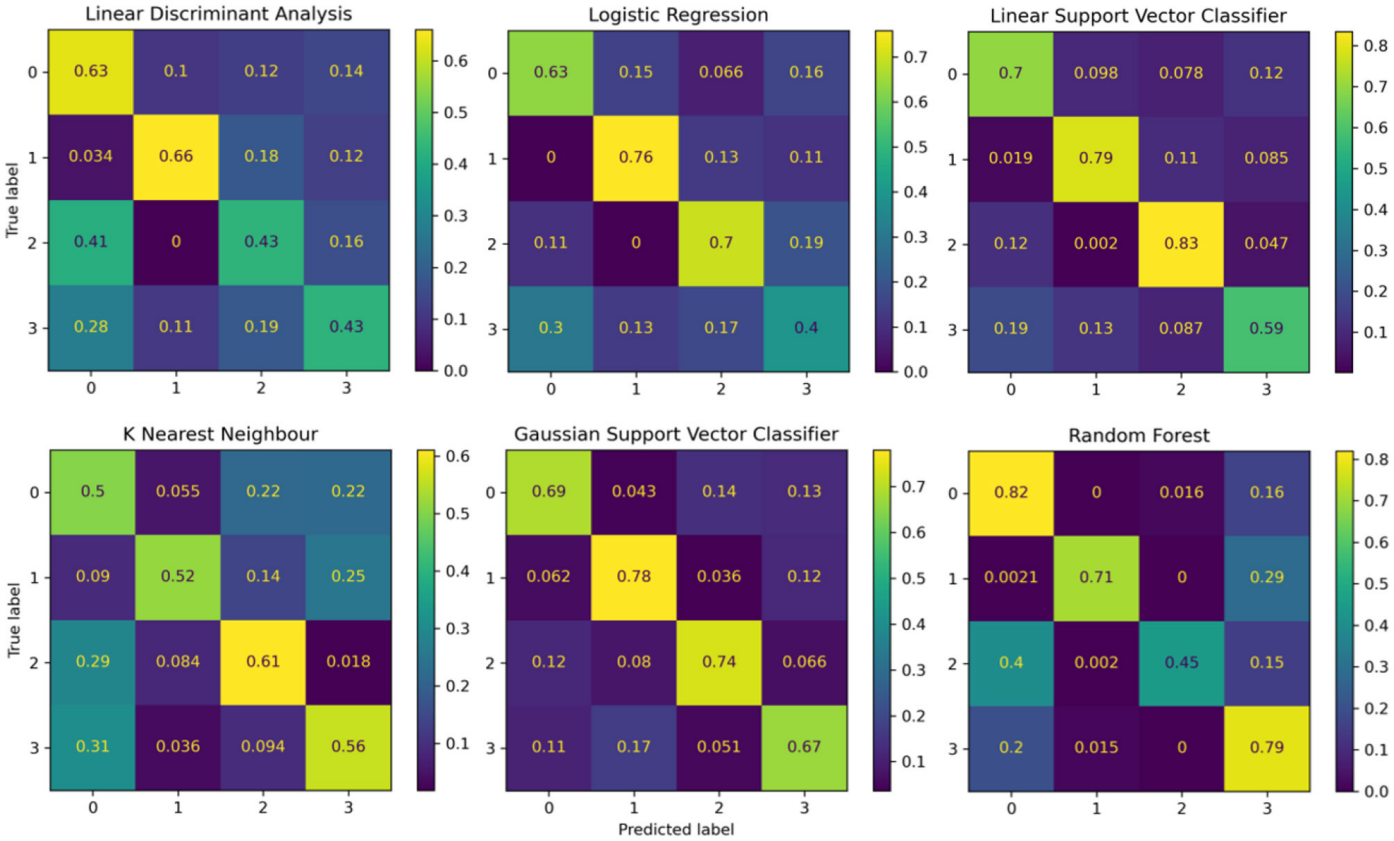
Confusion matrices summarising the models' performance. Each entry is presented as a percentage normalised to the total number of data samples. The vertical axis present the ground truth labels and the horizontal axis shows the predicted label determined by each class, (0) low pain cold, (1) low pain heat, (2) high pain cold, and (3) high pain heat.

The performance of the reference models using classical machine learning techniques and hand-crafted features is presented in Table 2. These results are presented based on four standard performance evaluation metrics (please refer to Section 2.7 for more details): accuracy, precision, recall, and F1 scores. The baseline models had contrasting results, LDA exhibited the lowest accuracy out of the three reference models with an overall accuracy of 53.5 ± 4.4. The KNN model presented the second lowest performance with an accuracy of 59.7 ± 2.1. The LR model presented an accuracy of 62.1 ± 2.6. The RF classifier exhibited an accuracy of 68.3 ± 3.3. The GSVM presented the second highest accuracy among all classifiers, with an accuracy of 71.3 ± 4.6. The LSVM model presented the best performance with an overall accuracy of 72.93 ± 3.8 in our multiclass problem. In addition, the confusion matrices for all reference models is presented in Figure 8. The confusion matrices revealed that in most cases, the classifiers misclassified instances of class 3 (high pain heat) with the other classes, resulting in a lower true positive rate for class 3.

**Table 2:** Comparison of the average (±std) performance metrics of the baseline models, LDA, LR, LSVM, KNN, GSVM, and RF.

**Model**	**Accuracy**	**F1-score**	**Precision**	**Recall**
LDA	53.6 ± 4.4	53.4 ± 4.3	54.7 ± 4.3	53.6 ± 4.4
LR	62.1 ± 2.6	61.6 ± 2.6	61.5 ± 2.9	60.0 ± 2.6
LSVM	72.9 ± 3.8	72.6 ± 3.9	72.8 ± 4.0	71.8 ± 3.8
KNN	59.7 ± 2.1	52.9 ± 4.3	65.0 ± 3.7	58.9 ± 2.3
GSVM	71.3 ± 4.6	71.1 ± 4.7	72.4 ± 4.5	71.6 ± 4.3
RF	68.3 ± 3.3	68.4 ± 3.5	76.5 ± 2.2	68.2 ± 3.3

All results are presented in percentage (%).

### 3.3 Performance of the deep learning models

**Figure 9:**
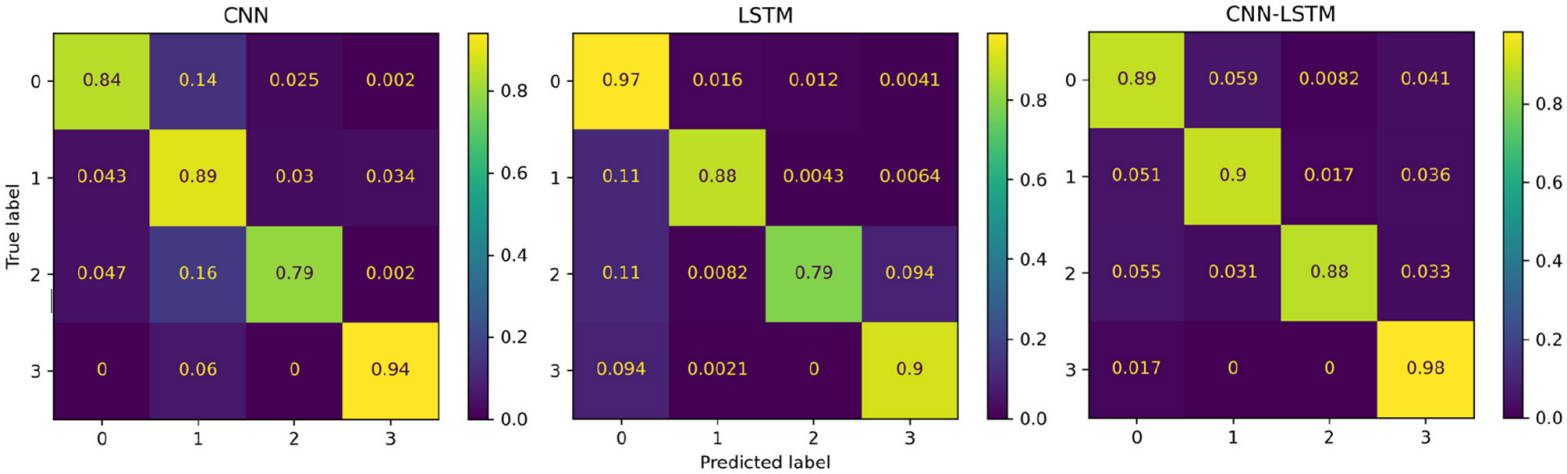
Confusion matrices summarising the deep learning models' performance. Each entry is presented as a percentage normalised to the total number of data samples. The vertical axis present the ground truth labels and the horizontal axis shows the predicted label determined by each class, (0) low pain cold, (1) low pain heat, (2) high pain cold, and (3) high pain heat.

The performance of the proposed deep learning models are presented in Table 3. The individual CNN and LSTM methods exhibited a similar performance with an accuracy of 86.4%±16.8 and 88.4%±21.1, respectively. The hybrid CNN-LSTM architecture exhibited the highest accuracy in our experimental conditions with 91.2%±11.7. Overall, the CNN-LSTM model presented the best results in all of the performance metrics in this study. In general, all deep learning models exhibited acceptable results to classify the different levels of pain stimulation without the need of featured engineering and only using minimally preprocessed fNIRS data. The confusion matrices for the three deep learning models is presented in Figure 9. Similar to the reference models, the confusion matrices indicate that the deep learning models often misclassified instances of class 3 (high pain heat) with other classes, leading to a reduced true positive rate for class 3.

**Table 3:** Comparison of the average (±std) performance metrics of the proposed CNN, LSTM, and CNN-LSTM models.

**Model**	**Accuracy**	**F1-score**	**Precision**	**Recall**
CNN	86.4 ± 16.8	85.1 ± 19.1	89.7 ± 13.9	86.1 ± 13.4
LSTM	88.4 ± 21.1	86.4 ± 24.95	89.8 ± 16.4	88.1 ± 20.8
CNN-LSTM	91.2 ± 11.7	91.0 ± 12.1	92.2 ± 10.1	90.8 ± 11.3

All results are presented in percentage (%).

Finally, the statistical analysis using the accuracy results was carried out. The ANOVA results showed that the *p*-value (*p* = 2.2811*e*−12, F-statistic = 13.69) is smaller than the significance level (0.05), which indicates to reject the null hypothesis that there are no significant differences in performance between the models. The *post-hoc* test (refer to Table 4) exhibited the models that presented statistically significant differences with respect to the accuracy results. From these results, it is evident that the proposed deep learning models exhibited significantly better performance against the baseline models (LR, LDA, SVM, KNN, GSVM, RF). In particular, the CNN-LSTM model presented statistically significant differences against all the reference models, LDA (*p* = 0.0), LR (*p* = 0.0001), LSVM (*p* = 0.0145), KNN (*p* = 0.0), GSVM (*p* = 0.0056), RF (*p* = 0.001); while the individual CNN and LSTM exhibited significant differences against LDA (CNN *p* = 0.0, LSTM *p* = 0.0), LR (CNN *p* = 0.0001, LSTM *p* = 0.0), GSVM (LSTM *p* = 0.0306), RF (CNN *p* = 0.001, LSTM *p* = 0.0065). It is also evident that CNN, LSTM, and CNN-LSTM models perform equally well, as there are no statistically significant difference between them.

**Table 4:** *Post-hoc* results, using Tukey's test, based on the accuracy results of all deep learning models.

**Group 1**	**Group 2**	**Mean diff**.	***p* value**	**Lower**	**Upper**	**Reject**
CNN	CNN-LSTM	4.706	0.9907	-11.45	20.86	FALSE
CNN	GSVM	-15.13	0.0844	-31.29	1.02	FALSE
CNN	KNN	-26.67	**0.0**	-42.83	-10.51	TRUE
CNN	LDA	-31.71	**0.0**	-47.87	-15.55	TRUE
CNN	LR	-24.98	**0.0001**	-41.14	-8.82	TRUE
CNN	LSTM	1.92	1	-14.23	18.08	FALSE
CNN	LSVM	-13.63	0.1685	-29.79	2.52	FALSE
CNN	RF	-17.69	**0.0213**	-33.85	-1.53	TRUE
CNN-LSTM	GSVM	-19.84	**0.0056**	-36	-3.68	TRUE
CNN-LSTM	KNN	-31.38	**0.0**	-47.54	-15.22	TRUE
CNN-LSTM	LDA	-36.42	**0.0**	-52.58	-20.26	TRUE
CNN-LSTM	LR	-29.68	**0.0**	-45.84	-13.52	TRUE
CNN-LSTM	LSTM	-2.78	0.9998	-18.94	13.37	FALSE
CNN-LSTM	LSVM	-18.33	**0.0145**	-34.49	-2.17	TRUE
CNN-LSTM	RF	-22.39	**0.001**	-38.55	-6.23	TRUE
GSVM	KNN	-11.54	0.3687	-27.7	4.62	FALSE
GSVM	LDA	-16.58	**0.0398**	-32.74	-0.42	TRUE
GSVM	LR	-9.84	0.5876	-26	6.31	FALSE
GSVM	LSTM	17.05	**0.0306**	0.89	33.21	TRUE
GSVM	LSVM	1.5	1	-14.65	17.66	FALSE
GSVM	RF	-2.55	0.9999	-18.71	13.6	FALSE
KNN	LDA	-5.04	0.9854	-21.2	11.11	FALSE
KNN	LR	1.69	1	-14.46	17.85	FALSE
KNN	LSTM	28.59	**0.0**	12.43	44.75	TRUE
KNN	LSVM	13.04	0.2145	-3.11	29.2	FALSE
KNN	RF	8.98	0.6998	-7.17	25.14	FALSE
LDA	LR	6.73	0.9196	-9.42	22.89	FALSE
LDA	LSTM	33.63	**0.0**	17.47	49.79	TRUE
LDA	LSVM	18.08	**0.0168**	1.92	34.24	TRUE
LDA	RF	14.02	0.1418	-2.13	30.18	FALSE
LR	LSTM	26.9	**0.0**	10.74	43.06	TRUE
LR	LSVM	11.34	0.3917	-4.81	27.5	FALSE
LR	RF	7.29	0.8796	-8.87	23.45	FALSE
LSTM	LSVM	-15.55	0.0686	-31.71	0.6	FALSE
LSTM	RF	-19.61	**0.0065**	-35.77	-3.45	TRUE
LSVM	RF	-4.05	0.9966	-20.21	12.1	FALSE

Statistically significant differences between compared models are presented in bold text. Reject = TRUE indicates to reject the null hypothesis (*H*_*o*_) at the 5% significance level.

## 4 Discussion

This study explores the use of deep learning models for the objective assessment of pain using fNIRS data. In particular, we used deep learning as a method to reduce the level of subjectivity and domain knowledge in feature engineering. Also, we compared different deep learning models to propose a possible method for pain assessment. The results showed that the deep learning models exhibited favourable results to identify the four different types of pain using only fNIRS input data. In addition, among the deep learning models compared in this study, the combination of CNN and LSTM in a single hybrid model exhibited the highest performance in our problem setting.

One objective of this study was to explore the possibility to use deep learning to eliminate the need for manual feature extraction. In a previous study (Rojas et al., 2019), the use of different domains (time, frequency, and wavelet) to extract several features was explored, the results showed that using 69 features, with a combination of features from the three different domains, produced an accuracy of 88.41% using a Gaussian SVM. In another study, mean values of the oxy-HB signals were employed as feature extraction technique with an accuracy of 85.76% using a SVM with quadratic kernel (QSVM) and employing the mean values of the oxy-HB signals (Shamsi and Najafizadeh, 2020). In a study by Lopez-Martinez et al. (2019), 80 features were obtained from discretised values using the continuous wavelet transform with an accuracy of 69% using a Gaussian SVM. In a study by Zeng et al. (2023), different functional connectivity features based on global and local nodal measures were obtained, and after feature selection, the top seven most important features obtained an accuracy of 75.59% using logistic regression. Feature selection is a powerful technique that helps identify and eliminate irrelevant features, thus, classification performance can be improved. Recently, it has been suggested that feature selection could enhance the performance of deep learning methods (Chen et al., 2020); therefore, considering feature selection may be explored in our future work. In general, the explored deep learning models show comparable results without relying on subjective manually extracted features or domain expertise to define features, reducing human intervention that might bias the interpretation of data (Silberg and Manyika, 2019).

The second objective of this study was to compare different deep learning models and to identify a potential method for pain assessment directly using fNIRS data. In particular, the hybrid CNN-LSTM model showed the best results (accuracy = 91.2%), performing consistently better than the other models in most metrics. In addition, the CNN-LSTM architecture was the only model that exhibited statistically significant better performance than all three reference models (LDA, LR, SVM). The CNN model also showed good results (accuracy = 86.4%). It is to noteworthy that CNNs have had a significant impact in various domains since AlexNet (Krizhevsky et al., 2012) won the ImageNet competition in 2012. The LSTM network exhibited the lowest performance (accuracy = 88.4%). It is widely known that the challenge with LSTMs is their requirement for a substantial amount of data to train effectively. This may lead to poor performance as LSTMs may struggle to generalise well and may memorise noise in the training data (Siami-Namini et al., 2019). Overall, the proposed models (LSTM, CNN, CNN-LSTM) exhibited acceptable performance in our experimental conditions.

The combination of CNN and LSTM in a single architecture (CNN-LSTM) outperformed the other deep learning models in our experimental setting. This type of hybrid architecture has shown better performance than many other state-of-the-art models in many time series applications. In recent neuroimaging studies, this hybrid architecture has found applications such as, EEG data for screening of depression with an accuracy of 99% (Sharma et al., 2021), mental state monitoring (workload, vigilance, fatigue) using fNIRS with an accuracy of 85.9% (Mughal et al., 2021), or epileptic seizure recognition using EEG with an accuracy of 99.39% (Xu et al., 2020). In these applications, the CNN architectures serve the dual purpose of filtering out noise from the input data and extracting valuable information crucial for the final prediction; While LSTM architectures possess the capability to identify both short-term and long-term dependencies. In this CNN-LSTM approach, the CNN is employed to gradually extract higher-level features and identify spatial relationships between the sequences of observations, and then these features are fed into the LSTM model to learn temporal relationships of the data and predict based on that (Rojas et al., 2021). The basic idea of the application of this hybrid architecture is to exploit and combine the advantages of these two deep learning techniques.

We acknowledge that there are limitations of our study that deserve consideration. It is possible that participants who were included in this study had pathologies or other disorders that were unknown by the subjects and therefore unknown by the investigators, this may confound the pain measures obtained during our experiments. It is important to highlight this since it has been reported that some disorders (e.g., anxiety, mood, or eating disorders) affect considerably the perception and tolerance of pain (Pistoia et al., 2013), which may have altered the results of this study. In addition, we studied the performance of deep learning models to classify different levels of pain using fNIRS data and we collected fNIRS from a small number of participants (*n* = 18); while this preliminary results help us to identify relevant information about our experimental conditions, setup, and deep learning models, this limitation should be addressed by including a larger number of participants in our future research. Finally, direct comparisons with other studies in the literature are difficult due to their use of different experimental conditions, different neuroimaging methods (e.g., EEG, PET), number of subjects, data transformations, cleaning procedures, or heavy optimisation in the implemented learning models.

## 5 Conclusions

This study demonstrates that the use of fNIRS in combination with deep learning models is a possible tool for the assessment of pain in experimental conditions. Deep learning models have demonstrated their capacity to automatically extract features from fNIRS data and have the potential to offer an enhanced approach for pain assessment, which could prove valuable for non-verbal patients in future applications. The outcomes presented in this study contribute to the progression of understanding pain assessment through fNIRS as a diagnostic method, marking a significant stride towards establishing a physiologically-driven diagnosis of human pain. Such advancements not only hold promise for vulnerable populations unable to self-report pain but also have broader implications for the general population.

Future research will centre on leveraging various sensor modalities, including but not limited to heart rate, respiration, galvanic skin response, and electroencephalography, for pain assessment. Employing a multi-modal approach that employs data from diverse sensors holds the promise of enhancing the robustness and comprehensiveness of real-time human pain assessment. This integrated strategy aims to unveil potential interrelationships among different sensor modalities, offering mutual support in the presence of artefacts or signal quality challenges. Furthermore, the exploration of diverse multi-modal data fusion techniques will be instrumental in unlocking the full potential of multiple sensors in our forthcoming investigations.

## Data availability statement

The data that support the findings of this study are available from the corresponding author upon reasonable request.

## Ethics statement

The studies involving humans were approved by TMU Joint Institutional Review Board under contract number 201307010. The studies were conducted in accordance with the local legislation and institutional requirements. The participants provided their written informed consent to participate in this study.

## Author contributions

RF: Conceptualisation, Software, Data curation, Formal analysis, Investigation, Writing – original draft, Writing – review & editing. CJ: Formal analysis, Software, Writing – review & editing. GB: Formal analysis, Investigation, Methodology, Writing – review & editing. K-LO: Conceptualization, Formal analysis, Methodology, Resources, Supervision, Writing – review & editing.
